# The role of rumination in illness trajectories in youth: linking trans-diagnostic processes with clinical staging models

**DOI:** 10.1017/S0033291716001392

**Published:** 2016-06-29

**Authors:** A. B. Grierson, I. B. Hickie, S. L. Naismith, J. Scott

**Affiliations:** 1 Brain and Mind Centre, The University of Sydney, Sydney, Australia; 2 Charles Perkins Centre & Brain and Mind Centre, The University of Sydney, Sydney, Australia; 3 Academic Psychiatry, Institute of Neuroscience, Newcastle University, Newcastle upon Tyne, UK

**Keywords:** Brooding, clinical staging, mental disorders, rumination, trans-diagnostic, youth

## Abstract

Research in developmental psychopathology and clinical staging models has increasingly sought to identify trans-diagnostic biomarkers or neurocognitive deficits that may play a role in the onset and trajectory of mental disorders and could represent modifiable treatment targets. Less attention has been directed at the potential role of cognitive-emotional regulation processes such as ruminative response style. Maladaptive rumination (toxic brooding) is a known mediator of the association between gender and internalizing disorders in adolescents and is increased in individuals with a history of early adversity. Furthermore, rumination shows moderate levels of genetic heritability and is linked to abnormalities in neural networks associated with emotional regulation and executive functioning. This review explores the potential role of rumination in exacerbating the symptoms of alcohol and substance misuse, and bipolar and psychotic disorders during the peak age range for illness onset. Evidence shows that rumination not only amplifies levels of distress and suicidal ideation, but also extends physiological responses to stress, which may partly explain the high prevalence of physical and mental co-morbidity in youth presenting to mental health services. In summary, the normative developmental trajectory of rumination and its role in the evolution of mental disorders and physical illness demonstrates that rumination presents a detectable, modifiable trans-diagnostic risk factor in youth.

## Introduction

Developmental psychopathology explores illness trajectories from a life-course perspective, and as such it may be especially helpful for understanding the emergence of mental disorders in youth (Scott *et al.* 2013). In young people, a particular challenge is to identify deviations in basic psychological and/or biological processes that can explain why the initial clinical presentation may demonstrate concurrent mental, physical, and alcohol/substance misuse disorders and/or the longitudinal phenomenology may show heterotypic continuity over time (McGorry *et al.* 2006; Hickie *et al.* 2013; Scott *et al.* 2013).

Attempts to understand evolving patterns of signs and symptoms have increasingly focused on the application of dimensional approaches such as *clinical staging* and the *Research Domain Criteria* (*RDoC*) initiative (Insel *et al.* 2010; Sanislow *et al.* 2010; Hickie *et al.* 2013; McGorry *et al.* 2014). Both approaches highlight the need to consider psychopathology on a continuum (from normal experiences through to established clinical disorders) and emphasize that our understanding of liability and co-morbidity is more likely to be improved by examining putative underlying mechanisms that cut across current heterogeneous disorder categories (Baskin-Sommers & Foti, 2015). Implicit in these frameworks is the notion that identifying trans-diagnostic processes that translate into ‘modifiable factors’ is a critical step in the development of precision or personalized medicine for mental disorders (Insel *et al.* 2010; McGorry *et al.* 2014), and moreover, that clinical advances will only really be achieved by greater integration of behavioural neuroscience into the study of psychopathology (Sanislow *et al.* 2010). However, research to date has predominantly focused on ‘biomarkers’ (measurable biological characteristics) and/or neurocognition (‘cold circuits’) (McGorry *et al.* 2014). There have been fewer reviews that synthesize data on ‘hot cognitions’ (Simon *et al*. 2015), such as cognitive processing styles involved in emotional regulation.

Given the lack of reviews that focus on cognitive-emotional processes that may act as a trans-diagnostic risk factor in young people with emerging severe mental disorders, we explore whether a particular ‘coping style’, namely rumination (and particularly ‘toxic’ brooding), can be considered as a specific age- and stage-related mechanism for psychopathology in youth. Whilst rumination has been studied extensively, we particularly focus on its evolution and its role in shaping emotional responses to stress in childhood, adolescence and early adulthood, including the peak age of onset period for psychosis, mood disorders and alcohol/substance misuse (between the ages of about 15 and 25 years) (Kessler *et al.* 2005; Jones, 2013) and whether rumination is a mechanism that can also help explain why many disorders in young people show heterotypic continuity over time and related phenomena such as multi-finality and pluri-potentiality (Ehring & Watkins, 2008).

The paper begins by briefly discussing cognitive-emotional regulation and the concept of coping response style, and then reviews rumination from the perspective of theories about its evolution, its normal developmental trajectory and its role in the evolution of high prevalence disorders (such as anxiety and depression) that are often the early stage clinical phenotypes that co-occur with severe mental disorders in adolescence and early adulthood. Next, the paper explores the emerging evidence for links between rumination and alcohol and substance misuse, and bipolar and psychotic disorders. Finally, we briefly highlight the neurobiological underpinnings of rumination and how its putative role in physiological responses to stress may offer insights into the high prevalence of physical and mental co-morbidities in young people.

## Brief overview of cognitive-emotional regulation and rumination

Cognitive-emotional regulation (CER) encompasses any attempt to understand and shape the emotions experienced and how these are expressed (Gross & Munoz, 1995). This regulation can be implicit (automatic, unconscious) or explicit (deliberate, requiring conscious effort) (Gyurak *et al*. 2011). Conscious, effortful cognitive processes can be adaptive or maladaptive and include strategies such as reappraisal and rumination (Garnefski & Kraaij, 2007).

The most widely researched example of CER is the Response Styles Theory (RST; Nolen-Hoeksema, 1987). RST focuses on individual styles of processing emotion that emerge in reaction to stressors and identifies four main coping responses: distraction, problem solving, risk taking, and rumination (see Table 1). Problem solving and distraction are considered to be adaptive response styles as they can positively impact on outcomes and provide opportunities for positive reinforcement, which increases future positive emotions and decreases negative affect (Abela *et al*. 2007). Risk-taking is considered maladaptive as, although it may temporarily offer distraction from or avoidance of difficult situations or feelings, the ‘downstream’ effects are more often negative (Nolen-Hoeksema, 1987; Nolen-Hoeksema *et al*. 2008).

**Table 1.** Key elements of cognitive emotion regulation and response styles theory

Cognitive emotional regulation (Gross & Munoz, 1995; Gyurak *et al*. 2011)			
**Emotion regulation**		Process	Examples
Explicit	Deliberate and conscious, and demands some level of monitoring during implementation. It is performed with insight and awareness	A situation is selected, modified, attended to, and appraised to yield a certain set of emotional responses. This is conducted in accordance with immediate and long term goals	• Humour • Distraction
Implicit	Automatic and unconscious, and thus requires little effort. It is performed without monitoring, insight, or comprehension	Like explicit emotion regulation, a situation is selected, modified, attended to, and appraised to produce a set of emotional responses. However this is done without cognitive engagement in the emotion regulation process	• Suppression • Avoidance

Response style theory (Nolen-Hoeksema, 1987, 1991; Abela *et al.* 2007)			
**Response style**		*Cognitions/behaviours*
Rumination	Behaviours and thoughts that focus attention on one's depressive symptoms and on the implication of these symptoms	• Symptom focused cognitions • Isolating behaviours
Problem solving	Movement toward a desired end state by active engagement in strategizing to overcome an issue	• Deliberate cognitions • Suppression of distracting thoughts • Goal-directed behaviours
Distraction	Engaging in pleasant, engrossing, and positively reinforcing activities to divert attention from one's depressive symptoms	• Suppression of self-contemplation • Active and pleasurable behaviours
Risk taking	Behaviours that have the potential to be harmful or dangerous, but that may allow the individual to avoid engaging with painful or intense emotions	• Cognitive reappraisal of fear • Positive outcome expectancies • Active and instantly gratifying behaviours

Rumination can be viewed as a stable individual trait (Smith & Alloy, 2009) characterized by ‘the tendency to repetitively analyse one's problems, concerns and feelings of distress without taking actions to make positive changes’ (Nolen-Hoeksema, 1991, p. 569). Rumination appears to be a multifaceted construct, having adaptive and maladaptive components, often referred to as ‘reflective pondering’ and ‘toxic brooding’, respectively (Treynor *et al*. 2003). Treynor *et al*. (2003) define reflection as a purposeful turning inward to engage in cognitive problem solving to alleviate one's distress whilst brooding is defined as ‘passive comparison of one's current situation with some unachieved standard. Brooding is associated with the maintenance and exacerbation of negative mood states such as depression and dysphoria (Verhaeghan *et al*. 2014), and negative cognitive biases such as attentional engagement with negatively valanced stimuli (Joormann, 2004).

Several models of rumination confirm that it acts to amplify and prolong a prevailing mood state (see Smith & Alloy, 2009), increasing the likelihood that dysphoria will progress to depression, and can increase anxiety in individuals faced with threatening situations (Nolen-Hoeksema & Watkins, 2011). Negative cycles of rumination and dysphoria make it increasingly likely that an individual will focus on prior negative thoughts and experiences, which further impairs instrumental behaviour and effective problem solving (Brosschot *et al*. 2006; Nolen-Hoeksema & Watkins, 2011). In contrast, there is emerging evidence that positive self-focused attention (positive reflection or ‘basking’) can be associated with lower levels of anxiety or negative affect (Segerstrom *et al.* 2003; Feldman *et al*. 2008; Raes *et al.* 2012; Hou & Ng, 2014).

How ruminative thought processes, such as toxic brooding, emerge and the underpinning mechanisms have been addressed by several research groups. The most prominent models of rumination, like RST, propose that rumination emerges in reaction to emotions such as negative affect and sadness, and that it is the styles of processing and reacting to emotions that lead to adverse psychological and physical health consequences (Nolen-Hoeksema, 1991; Conway *et al.* 2000). Other models, such as the ‘goal progress theory’ or ‘goal conflict theory’ propose that rumination is a response to behavioural inhibition and failure to progress satisfactorily towards a goal, or inaction and increased negative cognitive rehearsal associated with conflictual strivings, rather than a reaction to mood state *per se* (Emmons & King, 1988; Martin *et al*. 1993). It is suggested that failure to progress toward a goal and act on competing desires inaugurate ruminative thinking, which then leads to aversive psychological consequences such as mental disorders (Jones *et al.* 2009; Einstein, 2014). There is evidence that individuals who fail to progress toward goal states have lower wellbeing (Emmons & King, 1988), that high levels of goal failure combined with maladaptive rumination (brooding) are associated with more depressive symptoms (Jones *et al.* 2009), and that rumination mediates the link between goal attainment and positive affect (McIntosh *et al*. 1995).

Intolerance of uncertainty (IU), defined as a cognitive bias that affects how a person interprets and responds to uncertain situations (Dugas *et al*. 2004) is another mechanism proposed to underpin ruminative thinking (de Jong-Meyer*et al*. 2009; Yook *et al.* 2010; Einstein, 2014). It is suggested that individuals with IU are more sensitive to stress and use maladaptive coping strategies, such as dysfunctional cognitive processes or safety behaviours such as rumination, in order to reduce uncertainty, which may amplify affective states (McIntosh & Martin, 1992). Studies of IU and rumination demonstrate that rumination completely mediates the relationship between IU and depression (Yook *et al.* 2010; Liao & Wei, 2011), but other cognitive biases such as worry did not show a mediation effect (Yook *et al.* 2010).

## Developmental trajectory of rumination

This section briefly examines evidence for the development of rumination over the life span, selecting three key topics to discuss: age, gender and early environment (including exposure to adversity).

### Age and gender

Rumination involves a process of cognitive appraisal and so requires some capacity for basic abstract thinking and formal operational thought, which usually become apparent during the transition from mid-childhood to early adolescence (Piaget, 1977; Cole *et al.* 2006). Population-based studies confirm that children and pre-adolescents show significantly lower levels of rumination than adolescents (Hampel & Petermann, 2005; Thompson *et al.* 2010). Interestingly, Sütterlin *et al.* (2012) showed a decline in rumination from early adulthood onwards, where the highest levels of ‘brooding’ occurred in individuals aged <25 years. Explanations of the age trajectory of rumination concentrate on the potential long-term impact of childhood coping skills development and experiences. For example, some investigators have suggested that a peak in toxic brooding during adolescence is linked to difficulties in coping with increased levels of normative stressors that occur in this age range especially in those who failed to acquire sufficient adaptive problem-solving skills at an earlier age (Compas, 1987).

Gender differences in rumination also emerge during adolescence, with levels increasing in a linear fashion for girls from 12 to 15 years of age onwards, but with limited increases in rumination in males during the post-pubertal period (Tamres Janicki & Helgeson, 2002; Jose & Ratcliffe, 2004; Jose & Brown, 2008). There are a number of theories as to why females may ruminate more than males, including gender-stereotyped coping style (Cox *et al*. 2010) and differences in self-perceptions of emotionality and the stressfulness of the environment (Rudolph & Hammen, 1999; Gohm, 2003; Hyde *et al*. 2008; Nolen-Hoeksema, 2012). Against the latter, a meta-analysis by Tamres *et al*. (2002) found that gender differences in rumination were independent of the appraisal of stressors. Interestingly, work on ‘pubertal timing’ suggests that early puberty predicted higher levels of interpersonal dependent events and enhanced the risk of dysphoric symptoms in boys and girls who had a more negative cognitive style and a lower level of emotional clarity (Hamilton *et al.* 2014*a*, *b*).

### Early environment

Some research indicates that future risk of becoming a ‘ruminator’ may be associated with early adversity, and rumination can be a learnt response style developed via parent modelling and communication (Hankin *et al.* 2009; Cox *et al*. 2010). Nolen-Hoeksema (1998) proposed that children who have little perceived control over their environment might be especially prone to becoming ruminators in adolescence. For example, pre-school exposure to ‘negative-submissive family expressivity’ has been shown to predict rumination in adolescence (Hilt *et al*. 2012), whilst exposure to ‘over-controlling’ parenting can undermine the child's sense of self-efficacy or mastery and contribute to the development of maladaptive coping strategies (Parker, 1983; Blatt & Homann, 1992) and a greater tendency to ruminate (Spasojevic & Alloy, 2002; Manfredi *et al.* 2011).

Neglect or abuse in childhood may shape a child's response to their environment, e.g. being vigilant to signs of threat and becoming passive to avoid aggravation in such situations, paying greater attention to negative stimuli, etc. (Macleod *et al.* 2002). In such circumstances, a child who feels helpless and socially and emotionally isolated may show reduced reliance on externally-oriented problem solving and an increased likelihood of engaging in solitary coping styles such as rumination (Browne & Finkelhor, 1986; Nolen-Hoeksema *et al*. 1994). Moreover, Pearson *et al*. suggested that childhood abuse increases the development of anxious attachments, which have been found to prospectively predict rumination (Pearson *et al*. 2011).

Several studies offer support for the association between early adversity and rumination. For example, in non-clinical samples of college students, individuals with a history of childhood sexual abuse showed higher levels of rumination about sadness and dysphoria than those without such a history (Conway *et al.* 2004). Likewise, clinical studies confirm an association between childhood sexual and emotional abuse and adult ruminative response style (e.g. Spasojevic & Alloy, 2002); however, trends were statistically significant for females but not males (although the latter might be due to limited statistical power). A recent study by O'Mahen *et al.* (2015) examined different experiences of childhood maltreatment in depressed and non-depressed pregnant women and suggested that childhood emotional neglect was related to behavioural avoidance whilst childhood emotional abuse was associated with rumination. Importantly, repeated assessment of nearly 1000 adolescents demonstrated that emotional abuse by parents or peers was an antecedent of negative coping and cognitive styles, and, as elaborated below, brooding mediated the association between abuse and future depressive symptoms (Padilla & Calvete, 2014).

It is important to note that all these adverse experiences may be linked by an overarching issue, namely that they reduce the capacity of a child or adolescent to engage in effective and balanced control of their multiple needs; this in turn may exacerbate feelings of hopelessness, especially if they are living in unsupportive environments (Mansell & Carey, 2013). Furthermore, these elements may increase the long-term risk of developing psychological problems and mental disorders in the future (Powers, 1973).

## Rumination and psychopathology

**Fig. 1.**
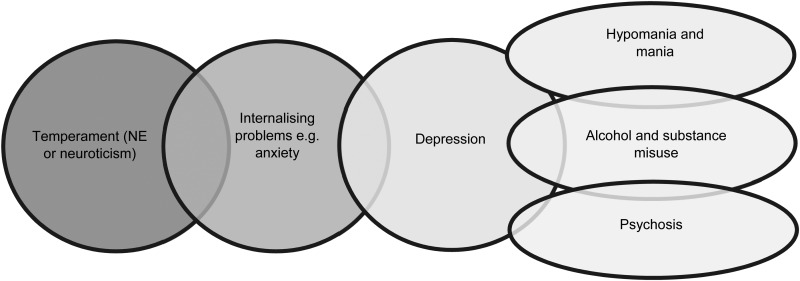
Representation of clinical stages of mental disorders beginning with early childhood temperament through to anxiety, depression, and then severe mental disorders (with peak age of onset in late adolescence/early adulthood).

The increased interest in early intervention in youth with severe mental disorders has led to greater awareness of illness trajectories, in particular that preschool temperament or personality traits can be risk markers for psychopathology such as internalizing or anxiety problems in childhood, which may precede the onset of depressive symptoms or disorders and be the antecedents of later psychopathology (see Fig. 1). Not all individuals show progression from high prevalence disorders (e.g. anxiety, depression) to the lower prevalence disorders (e.g. psychosis, bipolar disorders), which is a developmental pattern that mimics a clinical staging model (McGorry *et al.* 2006). Clinical staging involves a detailed assessment of where an individual exists on a continuum of disorder progression from stage 0 (an at-risk but asymptomatic state) through to stage IV (late or end-stage disease) (McGorry *et al.* 2006; Hickie *et al*. 2013; McGorry *et al.* 2014), and it is used routinely to describe disease progression in general medicine, e.g. for heart disease or diabetes. If this is a plausible approach for mental disorders, then trans-diagnostic mechanisms should be identifiable across stages and over time both within and between individuals.

### Temperament, rumination and future psychopathology

Temperament can be broadly defined as biologically based, relatively stable individual differences in emotions, behaviours, and cognitions (Rothbart & Bates, 2006). Negative emotionality (NE) is an infant temperamental style that is widely associated with psychopathology in adolescence, with high NE individuals experiencing negative emotions more frequently and intensely than their peers (Belsky *et al*. 1996), and are more likely to exhibit depression (Mezulis *et al*. 2011), anxiety (Hudson & Rapee, 2004), and/or substance abuse (Allen & Gabbay, 2013).

A prospective longitudinal study from birth to adolescence, found that rumination significantly mediated the association between NE in infancy and depressive symptoms at age 15 years (Mezulis *et al*. 2011). Consistent with other studies (e.g. Verstraeten *et al.* 2009), Mezulis *et al*. (2011) noted that the association was stronger among adolescent girls, although a further study by the same group highlighted that high levels of depressive symptoms at age 11 were associated with both early puberty and high levels of NE in boys (Mezulis *et al.* 2014).

Neuroticism is a similar but narrower construct than NE (Eysenck & Eysenck, 1985). Like NE, ruminative response style has been shown to fully or partially mediate the association between neuroticism and anxiety and depression in adolescents and young adults (Muris *et al*. 2005; Kuyken *et al.* 2006; Roelofs *et al.* 2008).

### Anxiety and depression

About 50% of individuals with anxiety disorders experience their first symptoms by the age of 11 (Kessler *et al.* 2005). A number of studies demonstrate that childhood anxiety and/or internalizing symptoms may predict adolescent depressive symptoms/episodes (Last *et al*. 1997; Horn & Wuyek, 2010), whilst anxiety disorders in early adolescence significantly predict first onset of a major depressive episode (MDE; Bittner *et al.* 2004). Rates of depressive disorders increase markedly during mid to late adolescence with about 20% of adolescents experiencing a MDE (Lewinsohn *et al*. 1998), and concurrent depression and anxiety occurs significantly more frequently in adolescence compared to childhood (Lamers *et al.* 2011).

Studies suggest that there is a robust relationship between rumination and the development of internalizing problems (such as withdrawal, anxiety and depression) but not externalizing problems (such as aggression, hyperactivity, etc.) in childhood (Garnefski *et al*. 2005). Rumination is also significantly associated with the development and maintenance of anxiety symptoms in adolescents (e.g. Garnefski *et al*. 2001, 2002; Tan *et al.* 2012; Jose *et al.* 2012; Jose & Weir, 2013), and increases the risk of developing anxiety problems in early adolescence (Tan *et al.* 2012). Furthermore, in a mid-teen sample, social anxiety (a recognized antecedent of severe mental disorders) directly predicted higher levels of rumination over time, especially in females (Jose *et al*. 2012).

Stressful life events and experiences such as bullying are potent risk factors for the development of internalizing symptoms (e.g. McLaughlin *et al*. 2009). In cross-sectional and prospective studies, McLaughlin and colleagues (e.g. McLaughlin & Hatzenbuehler, 2009; McLaughlin & Nolen-Hoeksema, 2012) demonstrated that rumination fully mediated the relationship between stressful life events and anxiety and depression among adolescents, but only partially mediated the association in adults. A large-scale study by Michl *et al.* (2013) confirmed that rumination is a key mediator between stress and anxiety and depressive phenomena in adolescents.

Independently for depression, there is a vast literature that indicates that rumination is significantly associated with depression onset, maintenance, and relapse, especially during adolescence (e.g. Wood *et al.* 1990; Nolen-Hoeksema, 2000; Michalak *et al.* 2012). A meta-analysis demonstrated that the higher levels of rumination in females compared to males significantly mediated the increased rates of depression that becomes apparent post-puberty (Aldao & Nolen-Hoeksema, 2012). Broderick (1998) suggests that adolescents use rumination to deal with family, academic, and peer group problems, which become increasingly more stressful and prominent during the adolescent period. However, as ruminative response style exacerbates rather than reduces the negative outcomes of stressful life events, it increases the likelihood of depressive outcomes (Schwartz & Koenig, 1996; Broderick, 1998; Kraaij *et al.* 2003). Furthermore, a study of an ethnically diverse sample of over 1000 college students demonstrated that individuals who brood in response to negative life events may be vulnerable to thinking about suicide, and that whilst level of suicidal ideation was partly explained by severity of symptoms of depression, it was also directly related to brooding itself (Chan *et al*. 2009).

### Alcohol and substance misuse

The prevalence of alcohol use disorders (AUD) and substance use disorders (SUD) in adolescents range from about 5–10% (Merikangas *et al.* 2010; Merikangas & McClair, 2012). Approximately 40% of individuals with an AUD and just below 50% of those with SUD experienced their first symptoms of harmful use before 19 years (Helzer *et al*. 1991; Dennis *et al.* 2002).

In clinical settings, 70–80% of adolescents with AUD and SUD have a co-morbid mental disorder (Ḳaminer & Bukstein, 2008), including anxiety, depression, psychosis or bipolar disorders. Studies indicate that rumination increases the risk for harmful alcohol and/or substance use (e.g. Nolen-Hoeksema & Harrell, 2002; Caselli *et al.* 2008; Caselli *et al.* 2010), particularly in female adolescents where rumination predicted the onset of substance abuse (and of bulimia), and predicted a future increase in substance abuse symptoms over a 4-year period (Nolen-Hoeksema *et al.* 2007).

The association between rumination and alcohol and substance misuse may be direct or indirect. For example, some studies of adolescents suggest that depression increases rumination, which in turn leads to increased substance use, as demonstrated by the association between brooding and marijuana use (Adrian *et al.* 2014). Skitch & Abela (2008) showed that ruminative brooding predicted substance abuse for up to 18 weeks following negative events, and that this effect was exacerbated in older adolescents with an existing MDE. Other research supports a direct association (e.g. Willem *et al.* 2011), reporting that, independent of depression, adolescents with lower reflective pondering demonstrated higher drug consumption whilst higher brooding was associated with more problematic substance use.

### Bipolar disorders (BD)

The peak age of onset for BD is late adolescence and early adulthood, although most individuals experience one or more MDE, brief hypomania or sub-threshold mania before syndromal episodes of hypomania or mania occur (Duffy *et al.* 2009; Douglas & Scott, 2014). Those individuals with depression who may be at highest risk of transition to BD often show cyclothymic or hypomanic personality traits or have a family history of bipolar disorders (Duffy *et al.* 2009; Geoffroy *et al*. 2013; Bechdolf *et al.* 2014).

There is limited research on rumination and mania in adolescents and young adults, but studies in middle-aged adults or mixed samples of younger and older adults show that rumination is present in both the depressed and (hypo)manic phases of BD, is increased in unaffected first degree relatives compared to healthy controls, and that increased rumination predicts relapse and/or worse course of illness (Johnson *et al*. 2008; Van der Gucht *et al.* 2009; Green *et al.* 2011; Ghaznavi & Deckersbach, 2012).

A study of university students (mean age 22 years) (Thomas & Bentall, 2002), reported that ruminative response style was positively correlated with scores on the Hypomanic Personality Scale (Eckblad & Chapman, 1986). A second study of undergraduate students (mean age 19 years) by the same group found that rumination was independently associated with both depression and hypomania symptom scores (Knowles *et al.* 2005), and that risk-taking behaviours were also increased in association with rumination, especially in males. Knowles *et al.* (2005) hypothesized that rumination may exacerbate depressive symptoms whilst some individuals make active attempts to avoid negative or intense mood states by engaging in high-risk activities, which may be linked to hypomanic or manic symptoms (Van der Gucht *et al.* 2009; Weiss *et al*. 2015).

Studies of individuals at high risk of BD or the offspring of BD parents demonstrate early evidence of deficits in CER (e.g. Nijjar *et al*. 2014; Van Rheene *et al*. 2015). In a study of response styles, Jones *et al.* (2006) found that offspring with current or past evidence of psychopathology (affected offspring) and unaffected offspring had higher levels of rumination than age- and gender-matched controls. Pavlickova *et al*. (2014) demonstrated that, compared to offspring of healthy parents, affected offspring of bipolar parents showed significantly increased levels of rumination and of hypomanic cognitions compared to unaffected offspring or healthy controls. An experiential sampling study by the same group examined the inter-relationships between mood, self-esteem and response styles over 6 days (Pavlickova *et al.* 2015). Interestingly, increased negative as well as positive mood resulted in greater rumination in offspring and controls, and low self-esteem triggered greater risk-taking in the bipolar offspring group, while negative affect instigated increased active coping in the control group. Additionally, as in studies of depression, CER impairments such as increased ruminative response style are associated with suicidal ideation in young people with emerging bipolar disorders (Stange *et al.* 2015).

### Psychosis

The typical age of onset for psychotic disorders is late adolescence or early twenties, being slightly later in females than males (Hafner *et al.* 1994; Gogtay *et al.* 2011). Several studies report that anxiety or depression in childhood or adolescence may precede the onset of psychosis (Baynes *et al.* 2000; Mulholland & Cooper, 2000; Maggini & Raballo, 2006). Research in groups at high risk of psychosis indicate that transition is common in those who have experienced brief periods of psychosis or psychotic-like symptoms (Yung *et al.* 2006), whilst the presence of depression and/or anxiety is associated with increased suicidality, self-harm, disorganized behaviour, disorganized speech, and anhedonia (Fusar-Poli *et al.* 2014).

Rumination has been shown to be associated with hallucination-proneness and a range of mild anomalous experiences including feelings of unreality, perceptual alterations, and temporal disintegration (Jones & Fernyhough, 2009; Freeman *et al.* 2013). Experiential sampling in young adults with psychosis demonstrates that antecedent rumination and worry predict persecutory delusions and auditory hallucinations, and that rumination predicted the level of distress associated with these psychotic experiences (Hartley *et al.* 2014). Also, Halari *et al.* (2009) demonstrated that rumination can be associated with negative symptoms such as stereotyped thinking and emotional withdrawal.

As ruminative response style overlaps with perseverative thinking, it could indicate that rumination in psychosis is associated with underlying psychotic or dysphoric (anxiety/depressive) dimensions (Cernis *et al.* 2015). Theoretically, rumination might also help explain why some young people who experience psychotic symptoms become distressed and seek help, whilst others do not (Van Nierop *et al.* 2012; Rapado-Castro *et al.* 2015).

Lastly, in a cross-sectional study of psychotic inpatients with and without suicidality (*n* = 2383; mean age at onset 25 years), ruminative thinking, social withdrawal, and lack of activity were all associated with increased suicidality (Ahrens & Linden, 1996).

## Neurobiological underpinnings of rumination

Neurobiological factors play a key role in the activation and maintenance of cognitive-emotional processes. This section briefly examines a selection of the developing research on the associations between rumination in young people and genetics, neuropsychology, physiology, sleep and physical health.

### Genes

There are reasons to consider the role of genetics in the development of rumination. As noted, rumination may mediate the association between temperamental styles that are heritable (e.g. NE, neuroticism) and depression. Furthermore, children at high risk of depression (e.g. offspring of mothers with a history of MDE) show higher levels of rumination than controls (Gibb *et al.* 2012). A small number of twin studies have examined genetic and environmental influences (shared and non-shared elements) on rumination, brooding and mood disorders. In a Chinese study of adolescent twins, the heritability of depression (30–42%) was modest, but genetic effects accounted for 24% of the variation in adolescent rumination, and genetics mediated the relationship between rumination and depression (Chen & Li, 2013). In a US study of adolescent twins aged 12–14 years, heritability accounted for 21% of the variation in adolescent brooding, highlighting again that although the heritable influences were modest, they accounted for the majority of the relationship between brooding and depression (h^2^ = 0.62) (Moore *et al.* 2013). Preliminary data from a UK study of BD in twins and siblings showed that ruminative response style had a low to moderate heritability with 23% of the total variance accounted for by additive genetic effects (Beards *et al.* 2012).

Certain polymorphisms of the BDNF gene (a protein involved in the growth of new and existing neurons and synapses) have also been found to be linked to rumination (Beevers *et al*. 2009). Results suggest that individuals with the Val^66^Met BDNF gene polymorphism are significantly more likely to ruminate than individuals without the gene. This is consistent with recent research that has found an association between the BDNF gene Val^66^Met polymorphism, rumination, and depression (Hilt *et al.* 2007), where analyses suggested that rumination mediated the relationship between the BDNF polymorphism and depressive symptoms.

### Neuropsychology

A number of cognitive processes are activated when engaging in rumination, in particular attention, memory, and self-referential processing (Lyubomirsky *et al.* 1998). Investigators have reported that rumination generates difficulties in controlling the entry of irrelevant information into working memory (WM) and short-term memory (STM) (Joormann, 2004; Goeleven *et al.* 2006; Joormann *et al*. 2010), or the removal of negative self-relevant information from WM and STM storage (Joormann & Gotlib, 2008). Ruminators also show general deficits in the ability to shift attention from unhelpful to helpful strategies (Davis & Nolen-Hoeksema, 2000). Futhermore, a study of over 200 adolescents demonstrated that engaging in ruminative thoughts consumes cognitive resources that would otherwise be allocated towards difficult tests of executive functioning (the resource allocation hypothesis). In contrast, no evidence was found to support the notion that lower levels of executive functioning at baseline predicted levels of rumination or depressive symptoms at follow-up (Connolly *et al.* 2014).

Functional magnetic resonance imaging (fMRI) shows that rumination is positively correlated with an increased activation of the medial prefrontal cortex and amygdala (Ray *et al.* 2005), and adults who ruminate regularly (e.g. dysphoric or depressed patients) show high connectivity between these structures (Gotlib & Hamilton, 2008). Ruminators also engage in higher rates of self-referential processing compared to their non-ruminative counterparts (Gotlib & Joormann, 2010); a process that can be examined via neuroimaging of the default mode network (DMN) (Ochsner & Gross, 2005; Schmitz & Johnson, 2006). In non-ruminators, activation of DMN brain regions is reduced after performing non-self-referential (i.e. goal-directed) activities during off-task or rest periods, suggesting an ability to lose one's self in the work (Sheline *et al.* 2009). In contrast, high ruminators show high connectivity between DMN regions during these rest periods, reflecting an inability to suppress self-referential thoughts during off-task times. Berman *et al.* (2011*b*) noted that in young adults (mean age 22 years) these connectivities were only found when participants engaged in brooding and not reflective pondering.

Marchetti *et al*. (2012) propose that an imbalance in the task positive (TP) and task negative (TN) elements of the DMN is the overarching neural mechanism involved in rumination. Studies of rumination in youth (Berman *et al.* 2011*a*) and of adult depressed cases *v*. controls (Hamilton *et al.* 2011) support the proposal that a TN-TP imbalance is associated with a failure to attenuate TN activity in the transition from rest to task periods and with toxic brooding.

A recent fMRI study compared healthy controls with unmedicated adolescents in remission from MDE (Jacobs *et al.* 2014) and showed that the remitted MDE adolescents exhibited hyper-connectivities within the DMN and between the DMN and salience networks (posterior cingulate cortex, subgenual anterior cingulate, and amygdala) and regions of the cognitive control network, which were related to rumination and sustained attention. However, it was not possible to conclude that the hyper-connectivities represented brain-based markers of traits (e.g. rumination), as it is plausible that the hyper-connectivities represent compensatory mechanisms in individuals with emerging mood disorders. Fewer studies examine CER in individuals aged 15–25 years with emerging bipolar disorders, although in mixed samples of younger and older adults it has been shown that individuals at increased risk of developing bipolar disorders are less successful at down-regulating amygdala activity and demonstrate inefficient use of reappraisal and distraction strategies (e.g. Heissler *et al.* 2014).

### Hypothalamic-pituitary-adrenal (HPA) axis and physiological studies

Several physiological studies have examined the relationship between rumination and heart rate variability. Key *et al.* (2008) demonstrated that heart rate variability in response to stress is more strongly associated with trait rather than state rumination in female college students.

A review of the relationship between rumination and cortisol identified that 13 of 17 studies were undertaken in children, students or young adults (Zoccola & Dickerson, 2012). Whilst higher levels of state rumination were consistently linked to increased cortisol concentrations, findings were less consistent for the physiological effects of stressors (basal cortisol levels or cortisol awakening response to naturally occurring or laboratory-induced stress). However, those studies that utilized social-evaluative stressor tasks generally showed that rumination predicted greater cortisol reactivity or delayed recovery. These findings are supported by a study using pupillary response to perceived rejection in depressed adolescents (Stone *et al.* 2016).

Gianferante *et al.* (2014) also showed that rumination in response to repeated stress predicted non-habituation of the HPA axis, which the authors suggest may offer a pathway linking rumination to negative health outcomes. Interestingly, this association may be bidirectional or attenuated by baseline physical status (Puterman *et al.* 2011). For example, Puterman *et al.* (2011) demonstrated that although ruminators experienced a more rapid initial increase in cortisol levels, greater HPA axis reactivity, and slower HPA axis recovery from stress, this profile was only significant in sedentary participants. In active participants, cortisol trajectories were equivalent in high and low ruminators. These findings are noteworthy given the evidence of increased sedentary lifestyles and lower physical activity in youth with mental disorders (even when medication-free) compared to their peers (Gehue *et al.* 2015; Vallarino *et al.* 2015).

### Sleep and circadian rhythms

Rumination may indirectly influence circadian rhythms through its association with increased cortisol production (Rea *et al.* 2010) and alterations in cortisol secretion patterns (for a review see Chan & Debono, 2010). Furthermore, studies of sleep in adolescents show rumination to be directly associated with poorer general sleep quality, longer time to fall asleep and more awakenings after sleep onset (Thomsen *et al.* 2003). Dysregulated sleep is associated with increased rumination compared to good sleepers with evidence that brooding is associated with fatigue, poor concentration and low mood (Carney *et al.* 2006). Zoccola *et al*. (2009) found that ruminating about a past stressor before bedtime predicted longer sleep onset latencies. This suggestion that post-stressor ruminative thought may predict delayed sleep onset is of particular interest as delayed sleep phase syndrome is a recognized marker of circadian disturbance that occurs more often in young compared to older adults (Robillard *et al.* 2014; Alloy *et al*. 2015; Steinan *et al.* 2015). Batterham *et al*. (2012) found that rumination and neuroticism mediated the relationship between self-reported sleep disturbance and new onset depressions in younger adults, but not the onset of generalized anxiety of panic disorders.

The above studies mainly relied on self-ratings rather than objective recordings of sleep. An exception is the study of university students (mean age about 20 years) by Pillai *et al.* (2014), which showed that nightly variations in pre-sleep rumination were predictive of significantly longer sleep onset latency (SOL) as recorded by actigraphy and by a self-report diary. It was estimated that, after controlling for baseline sleep disturbance and depressive symptoms, a one standard deviation increase on the pre-sleep rumination scale was associated with an approximately 7-min increase in actigraphy-based SOL.

### Physical health and immune system

Rumination has been found to be negatively associated with self-reported physical health, including higher levels of somatic complaints and lower general health (Lok & Bishop, 1999; Rector & Roger, 1996). In a longitudinal study of younger compared to older adults, Thomsen *et al.* (2004*a*) demonstrated that higher levels of rumination predicted poorer self-reported physical health and more somatic complaints in those aged 20–35 years only. High levels of rumination have been linked with immune functioning, but to date these associations have only been reported for some of the immune markers measured (numbers of leucocytes, lymphocytes, and polyclonal activation), and the findings were significant in older but not younger adults (Thomsen *et al.* 2004*b*).

**Fig. 2.**
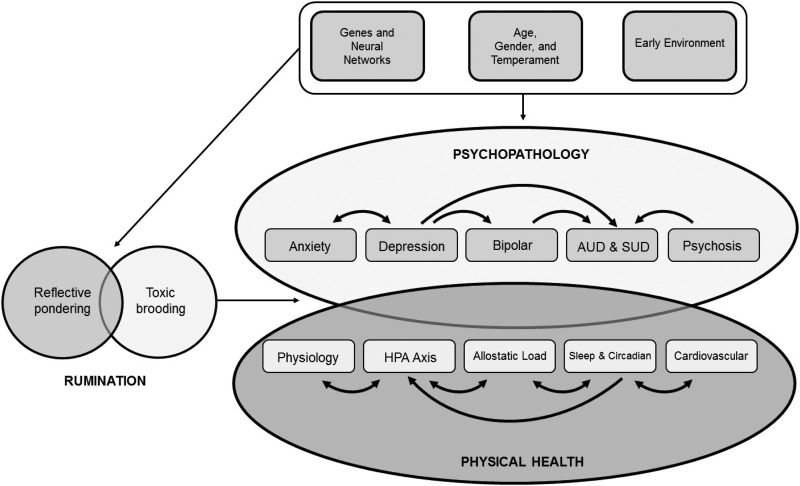
Model of rumination as a trans-diagnostic process impacting psychopathology and physical health, underpinned by genes and neural networks, age, gender, and temperament, and early environment. AUD, Alcohol use disorder; HPA, hypothalamic-pituitary-adrenal; SUD, substance use disorder.

Brosschot (2010) suggests that sustained cognitive representations of events (namely rumination and worry) can cause prolonged physiological activity, which may lead to intermediate pathogenic states (such as increased allostatic load) and finally to somatic disease. Glynn *et al*. (2002) found that ruminating about an emotional task resulted in increased blood pressure and delayed blood pressure recovery, which parallel findings reported in students with high levels of rumination and in adults (Bermúdez & Perez-Garcia, 1996; Neumann *et al.* 2001). Finally, although the co-morbidity between mood disorders and cardiovascular disease reported in young adults may be linked to medications or lifestyle factors (Goldstein *et al.* 2015), it is hypothesized that trait rumination may delay physiological recovery from acute stress and could act as a mechanism (Larsen & Christenfeld, 2009) (Fig. 2).

## Conclusions

This paper highlights several key aspects of rumination. First, we note that, of the two core components of rumination, it is the maladaptive (brooding) rather than the adaptive element (reflection or positive basking) that is consistently associated with the development of psychopathology. Also, the normative pattern of development of rumination parallels age and gender profiles associated with the typical evolution of internalizing disorders. Importantly, we highlight the association between the development of a ruminative response style and traumatic and abusive experiences in childhood, which are risk factors linked to a range of psychological problems in adolescence including mood and psychotic disorders. The evidence suggests that rumination is an underlying mechanism that contributes to a significant proportion of the explained variance between early adversity and later mental health problems. We do not claim that rumination is the only explanatory model, as other cognitive structures (e.g. beliefs and schemata) and processes have been implicated in the links between early trauma, CER and the development of psychopathology and acts of self-harm. Several studies demonstrate that these models and constructs may overlap and that there are links between early maladaptive schema, rumination and future symptoms of anxiety and depression in adolescents (Orue *et al*. 2014; Black & Pössel, 2015) or between early childhood trauma, over-general memory and high levels of ruminative thinking (Watkins & Teasdale, 2001; Williams *et al.* 2007). However, more research is needed to clarify the interactions between these different CER elements.

As reported in reviews of older adult populations, we confirm the importance of rumination in the evolution and maintenance of depression and highlight that in adolescence (but not always older adults) rumination may fully or partially mediate the relationship between childhood temperament and/or stressful events and the later onset of anxiety disorders. This sequence offers support for the notion of rumination being important in the longitudinal trajectory of the development of mental disorders and the notion that rumination plays a role in the transition between clinical stages. Our review differs from many previous reviews of rumination as a trans-diagnostic process operating across age groups and populations (Harvey *et al.* 2004; Ehring & Watkins, 2008; Watkins, 2009; Watkins & Nolen-Hoeksema, 2014), as we also examine the possible role of rumination in evolution of a problem from its sub-threshold phenomenology through to levels of harmful alcohol and substance use, bipolar and psychotic disorders that lead to help-seeking in an individual. Furthermore, we explore its putative influence on the trajectories of illness observed in this younger population and how similar processes may operate across physical and mental disorders. There is emerging evidence that rumination can be directly linked to the onset or maintenance of these problems in young adults, as well as existing evidence of indirect associations, with rumination exacerbating levels of anxiety or depressive symptoms, which in turn increase the distress that accompanies the symptoms or impede the ability to cope with those symptoms. It was also notable that rumination is frequently associated with suicidal ideation in depression, bipolar and psychotic disorders. We suggest that this is an important area for research, as the role of rumination in amplifying mood states and reducing flexibility in thinking styles and CER is under-explored in the age groups at the highest risk for onset of severe mental disorders or deliberate self-harm.

Evidence of the heritability of and neuropsychological pathways implicated in rumination is important as, for example, changes in the neural networks associated with CER may precede behavioural manifestations in unaffected high risk populations (Heissler *et al.* 2014). Critically, this review suggests ruminative response style is a putative shared mechanism for the development of sleep and physiological dysregulation and health problems in young adults with evolving mental disorders. This is relevant as attempts to explain physical and mental co-morbidities on the basis of factors such as the adverse physical effects of treatment or reduced daytime activity (post-onset of mental disorder) have proven to be overly simplistic and fail to take into account findings of the high levels of physical and mental co-morbidity in untreated, early stage cases. It appears that a ruminative response style may be associated with circadian and HPA disruptions, and its relationship to physical disease is not driven by greater reactivity in systems, but instead through extending activation and increasing allostatic load, which may increase the risks for cardiovascular or other damage (Larsen & Christenfeld, 2009). Indeed, Ottaviani *et al*. (2009) suggest that rumination represents an ‘autonomic phenotype’ because the associated autonomic dysregulation plays a role in the relationships between temperament, anxiety, depression and cardiovascular health.

In conclusion, this selective review of the association between rumination and the early clinical stages of mental disorders suggests that it is an important underlying trans-diagnostic process that operates in adolescents and young adults. Further, rumination shows a predictable developmental trajectory that is both detectable and modifiable. From the perspective of primary or early secondary prevention, modifying the maladaptive ‘toxic brooding’ component of rumination and substituting a more flexible response style that engenders a greater sense of self-control has the potential to reduce the onset or maintenance of problems such as depression and anxiety (Cook & Watkins, 2016), and may reduce the distress or severity of other disorders, such as psychosis and bipolar disorders (Vallarino *et al.* 2015; Scott, 2016). This review indicates that examination of rumination and other CER is an important, underexplored issue in trans-diagnostic and dimensional approaches to mental disorders, which is especially important given the apparent links to physical and well as mental health, and the prospects for modification of this risk factor.
